# Charles Arthur Wigan

**Published:** 1942

**Authors:** 


### CHARLES ARTHUR WIGAN, M.D. (Dur.), M.R.C.S., L.R.C.P.

Dr. Wigan, for many years a member of this Society, passed away at the ripe age of 86 at Portishead, where he had practised for fifty years.

The son of Dr. George Wigan, he was born in Australia, but went with his parents to Portishead when he was seven years of age.

He received his medical education at Durham University and Charing Cross Hospital, taking the diplomas of L.S.A. in 1881 and M.R.C.S. in 1882. He obtained the M.B. degree at Durham in 1883 and proceeded to the M.D. in 1888. After holding several resident appointments at Charing Cross he settled to practice at Portishead. Dr. Wigan had rendered much service to the town in addition to his medical work.

He was a member of the original Portishead Urban Council, which was appointed in 1892, and was the last surviving original member.

For twenty-three years he served on the Council, and was Chairman for eighteen of those years.

Dr. Wigan was a Justice of the Peace for Somerset.

For some years he was a member of St. Vincent Lodge of Freemasons in Bristol.

